# Interference effects in GaN high electron mobility transistor power amplifier induced by microwave pulses

**DOI:** 10.1038/s41598-022-21324-y

**Published:** 2022-10-08

**Authors:** Jingtao Zhao, Quanyou Chen, Chaoyang Chen, Zhidong Chen, Zhong Liu, Gang Zhao

**Affiliations:** 1 Science and Technology on High Power Microwave Laboratory, Institute of Applied Electronics, China Academy of Engineering Physics, Mianyang, 621900 China; 2 Key Laboratory of Science and Technology on Complex Electromagnetic Environment, China Academy of Engineering Physics, Mianyang, 621900 China; 3 Institute of Electronic Engineering of China Academy of Engineering Physics, Mianyang, 621999 China

**Keywords:** Applied physics, Electronics, photonics and device physics

## Abstract

Owing to the rapid development of wireless communication, radar and pulse power technology, the electromagnetic environment faced by electronic systems is increasingly complex and the intensity of electromagnetic field can be significant. In this study, a new interference phenomenon was observed when the microwave pulses were injected into the gallium nitride (GaN) high electron mobility transistor (HEMT) power amplifier through the output port. We investigated the relationship between the peak power of reverse injection microwave pulses and the duration or the amplitude of the interference by effect experiments. The interference duration could reach the magnitude of millisecond. Deep traps in GaN HEMT power amplifiers are proved to be the cause of this interference effects.

## Introduction

The unique material properties of gallium nitride (GaN), wide bandgap, high thermal conductivity, high breakdown voltage, high electron mobility and the device properties of GaN High electron mobility transistor (HEMT) namely low parasitic capacitance, low turn on resistance and high cut off frequencies make it a good choice for use in power amplifier (PA)^1–5^. In recent years, radar and electronic countermeasures systems based on GaN radio frequency (RF) devices have demonstrated longer transmission detection distance, sensitivity and durability, and obvious comprehensive performance advantages, which have strongly promoted the performance upgrade of military equipment. Development of next-generation communication system, i.e., fifth generation (5G) wireless communication will also bring revolutionary changes to the semiconductor industry. As the communication frequency band migrates to high frequency, both base stations and communication devices need RF devices that support high frequency performance. The advantages of GaN will gradually become prominent, making GaN a key technology in 5G^6–9^. However, with the rapid development of pulse power technology, the wide application of high-power radars and communication transmitters, the electromagnetic environment is becoming more and more complex, and the power density of the electromagnetic environment is also increasing, which makes the reliability of GaN-HEMT power amplifier inevitably be seriously threatened.

In this work, microwave pulses were injected into a GaN-HEMT power amplifier through the output port, and a new interference phenomenon was observed. The interference duration reached the order of milliseconds, which would cause a serious threat to the normal operation of the system.

## Experiment

**Figure 1:**
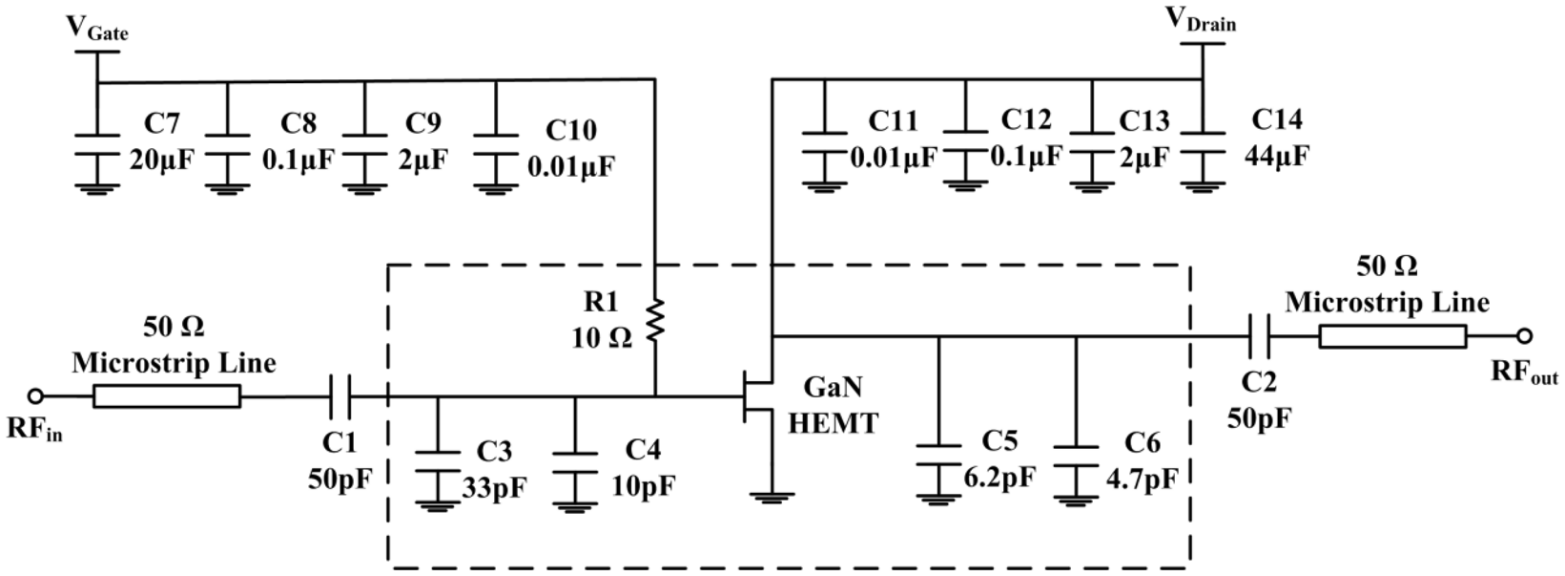
Structure of the power amplifier used in the study.

The power amplifier integrated circuit (IC) TGF2023-2-01 was fabricated by Qorvo using 0.25 μm high power GaN/SiC HEMT technology. The power amplifier, whose structure is shown in Fig. 1, is designed to work on S-band (2–4 GHz) of electromagnetic spectrum. The power amplifier can typically provide 38 dBm (about 6 watts) of saturated output power with power gain of 13.5 dB at 3 GHz. The maximum power added efficiency is 60.5%. *V*_Gate_ and *V*_Drain_ are the gate-to-source voltage and drain-to-source voltage, respectively. In this power amplifier, *V*_Gate_ is set to − 5 V and *V*_Drain_ is set to + 28 V.

**Figure 2:**
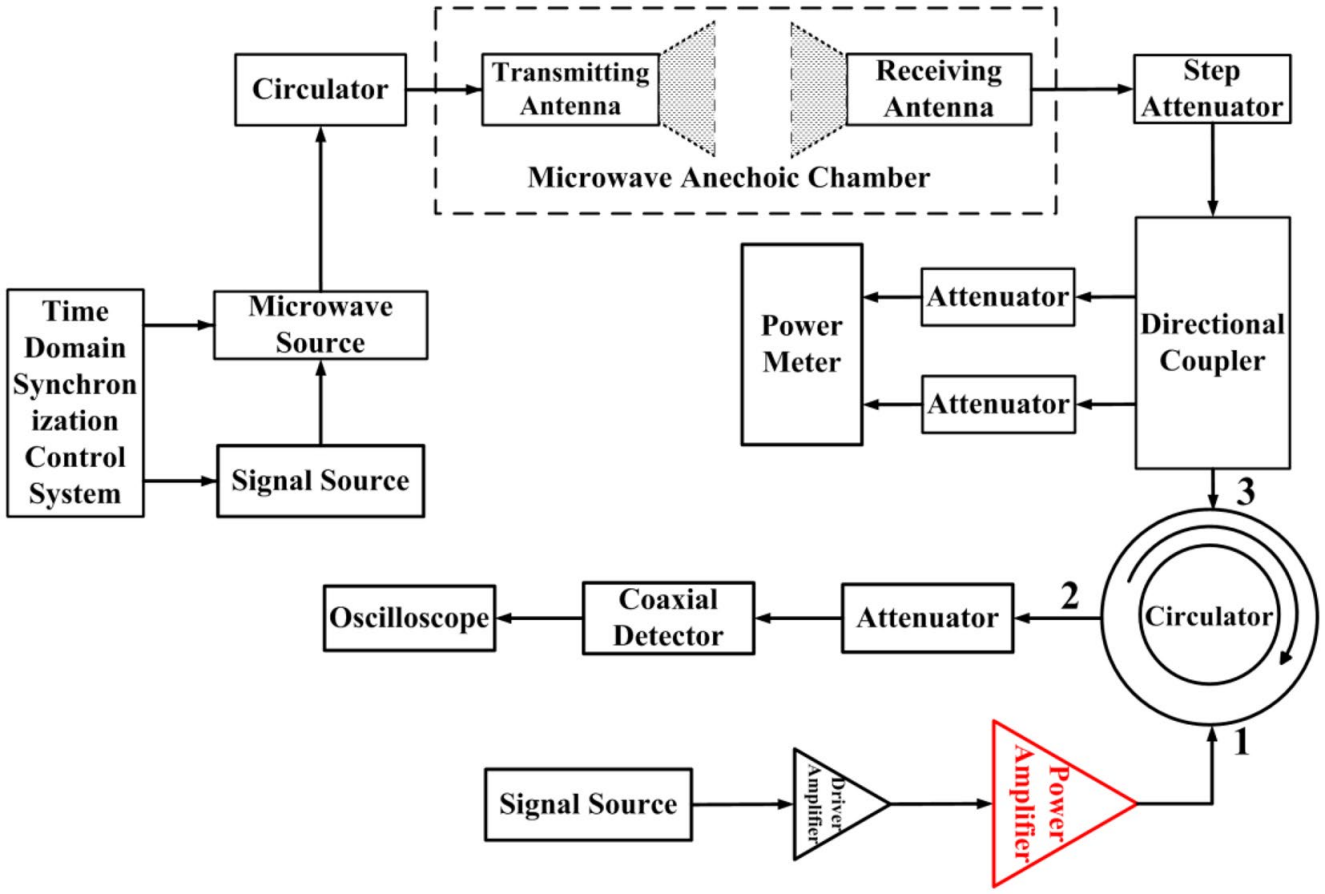
Schematic diagram of the experiment system employed for studying the interference effects in GaN-HEMT power amplifier induced by microwave pulses.

Figure 2 shows the schematic of the experiment system employed in our work for studying the interference effects in GaN-HEMT power amplifier induced by microwave pulses. The experiment system is designed based on the reception and injection mechanism of microwave radiation, and thus, it can be used to recreate practical application scenarios in a realistic manner. This system consists of a self-made microwave source system, several attenuators, circulator, directional coupler, RF power meter (R&S NRP2) and digital oscilloscope (LeCroy WavePro 640Zi). For our experiments, a series of microwave pulses are generated by the microwave source system, which can be changed gradually by tuning the step attenuator. Furthermore, a self-made time-domain synchronization control system and the signal source (Agilent E8257D) are used to control the pulse width, repetition frequency, and pulse number of the microwave pulses.

**Figure 3:**
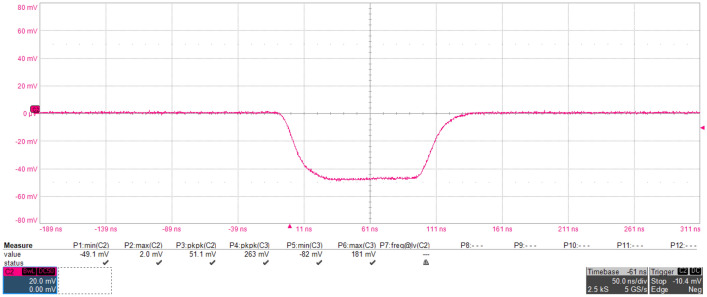
Typical waveform after demodulation injected into port 3 of the circulator.

**Figure 4:**
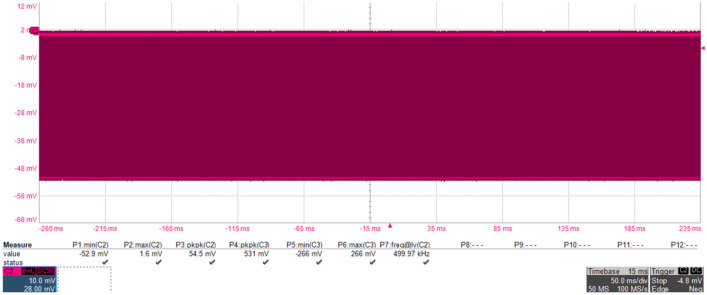
Output signals of the GaN-HEMT power amplifier in normal working state.

During the experiment, the signal source and the drive amplifier jointly drove the GaN-HEMT power amplifier to be in normal working state. The frequency of the signal source and microwave source injected were all 3 GHz. The operating frequencies for the transmitting and receiving antennas were 2.6 ~ 3.95 GHz and 1 ~ 18 GHz, respectively. Both antennas were vertical polarization and the distance between the antennas in the chamber was about 3 m. The power level at port 3 of the circulator injecting into the output of the PA was about 42.6 watts, and the true waveform can be regarded as a sine wave lasting 100 ns in the time domain. A typical waveform after demodulation injected into port 3 of the circulator was shown in Fig. 3. The saturated output power of the GaN-HEMT power amplifier was about 6 watts, and the output waveform of GaN-HEMT power amplifier was shown in Fig. 4. The coupling of the oscilloscope was set to DC 50 Ω and DC offset was not applied during the measurement. The modulated microwave pulses were injected into the GaN-HEMT power amplifier output port through the circulator (from port 3 to port 1), and the output signals (from port 1 to port 2) of the GaN-HEMT power amplifier were observed by the oscilloscope.

## Results and discussion

**Figure 5:**
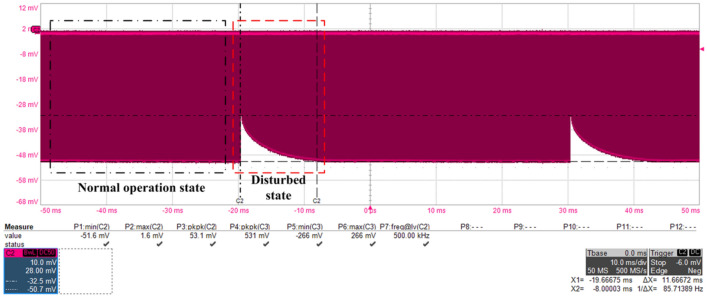
Output signals of the GaN-HEMT power amplifier observed by the oscilloscope when microwave pulses were injected into the output port.

The output waveform monitored by oscilloscope when microwave pulses were reverse injected into the output end of GaN-HEMT power amplifier was shown in Fig. 5. The injected microwave pulses had a peak power of 46 dBm (about 42.6 watts), a pulse width of 100 ns, and a repetition rate of 20 Hz. The pulse width of 500 ns and the period of 2 µs were the normal output waveforms of the GaN-HEMT power amplifier. As can be seen from the Fig. 5, microwave pulses of certain power intensity can cause interference effect in the output of GaN-HEMT power amplifier. The interference amplitude gradually weakened with the disappearance of microwave pulses. When the power amplifiers are used in radars or other RF systems, such a long time, high intensity of output interference, will affect the sensitivity and detection accuracy of the systems. If it is serious, the system cannot work properly.

In order to explore the causes of this interference effects, the output of voltage source *V*_Drain_ was monitored by oscilloscope while the microwave pulses were reverse injected the GaN-HEMT power amplifier. It was found that the output of power amplifier was interfered, while the output of the voltage source was remains stabled at + 28 V without any change. Therefore, the possibility of interference introduced by the voltage source was excluded. Since a large number of capacitors are used in the GaN-HEMT power amplifier for filtering the waves, in order to explore whether the interference is related to the capacitors, a large capacitor of 1000 µF was connected parallel to the right side of capacitor C6 and capacitor C14 respectively, and the experiment was repeated. It was found that the output interference phenomenon of the power amplifier was exactly the same as that without increasing the capacitors. The possibility that the interference was introduced by filter capacitors was also ruled out. Thus, the interference phenomenon can be basically determined to come from the GaN-HEMT itself.

**Figure 6:**
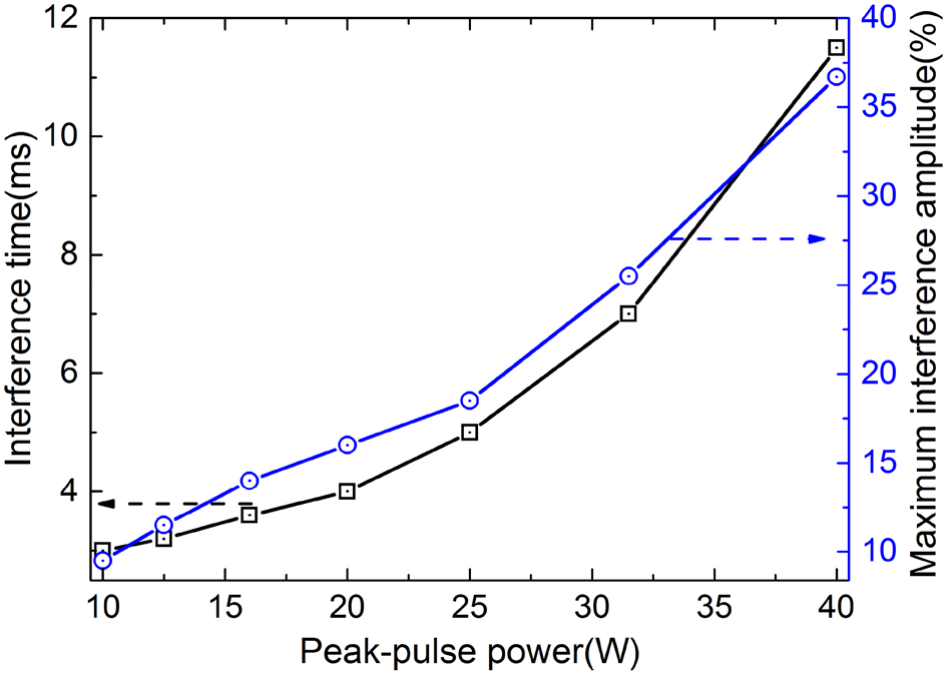
Relationships between the output interference time, maximum interference amplitude and the injected peak power of the microwave pulses.

In order to study the interference effects in GaN-HEMT power amplifier induced by microwave pulses systematically. By adjusting the adjustable attenuator, microwave pulses of different peak power were reversely injected into the GaN-HEMT power amplifier. The relationships between the output interference time, maximum interference amplitude and the injected peak power were shown in Fig. 6. As can be seen in Fig. 6, with the increase of of the peak power of the microwave pulses, the interference time and maximum amplitude are increasing. The interference time is on the order of milliseconds, much longer than the pulse width of the injected microwave pulses.

III-Nitrides are commonly grown on substrates with lattice mismatches. Growth is performed at high temperatures, which may be conducive to strong impurity contamination, high concentration of point defects and high strain caused by the difference in thermal expansion coefficients. All of these can result in high density of extended defects and centers with deep levels^10^. Deep traps remains an important problem and one of the major obstacles to the widespread use of GaN-HEMT. Trapping manifests itself in a variety of phenomena, such as lower output power at high frequencies, frequency dispersion, noise, gate lag and drain lag, high leakage currents, low breakdown voltage, device degradation under operation, high sub-threshold currents^11–19^.

**Figure 7:**
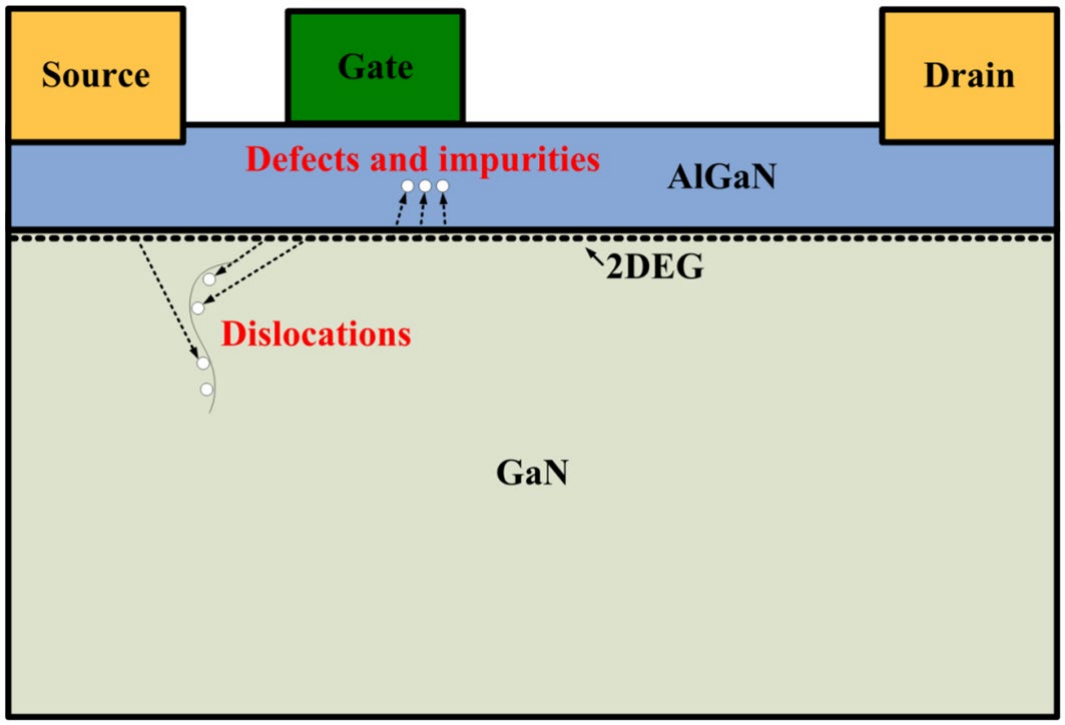
Schematic drawing of trapping in GaN HEMT induced by the microwave pulses.

When the microwave pulses are reverse-injected into the GaN-HEMT power amplifier from the output port, the strong electric field generated by the microwave coupling is directly loaded onto the drain of the GaN HEMT. The two-dimensional electron gas (2DEG) in the channel will gain a lot of energy from the microwave pulses and become high-energy electrons, which can cross the barrier and be captured by deep traps as shown in Fig. 7. The deep traps are related to native defects, impurities, and dislocations in general^10,20–29^. The trapping induced by deep traps results in a significant decrease in the 2DEG density within the channel, resulting in a decrease in the output current of the GaN HEMT and ultimately a large reduction in the output power of the power amplifier. Characteristic relaxation times of the deep traps measured for different transistor structures widely vary from microseconds to tens of milliseconds^10,30^, which is basically consistent with the interference time induced by microwave pulses. The higher the peak power of the microwave pulses, the stronger the electric field coupled to the drain of the GaN HEMT, and the more energy the 2DEG can capture and more deep traps trap more electrons. Therefore, the interference time and maximum interference amplitude increase with the increase of the peak power of the reverse injection microwave pulses.

The principle of GaAs pseudomorphic high electron mobility transistor (PHEMT) and GaN HEMT is similar, both are high electron mobility transistors working through the 2DEG. GaAs PHEMT has a much lower deep traps concentration than GaN HEMT due to its material properties and growth conditions^31–34^. To further verify the interference effect induced by deep trap, we selected a GaAs-pHEMT power amplifier (BW234) to perform the same experiment.

The GaAs-pHEMT power amplifier integrated circuit BW234 was fabricated by the 13th Research Institute Of China Electronics Technology Group Corporation. The power amplifier was also designed to work on S-band (2.7–3.5 GHz) of electromagnetic spectrum. The GaAs-pHEMT power amplifier can typically provide 40 dBm (about 10 watts) of saturated output power with power gain of 24 dB at 3 GHz. The maximum power added efficiency was 33%. *V*_Gate_ was set to − 0.7 V and *V*_Drain_ was set to + 8 V. Unlike the GaN HEMT, the peak power of the reverse injection microwave pulses was continuously increased, but no interference was found until the GaAs-PHEMT power amplifier was burned out. This supplementary experiment further confirmed that the interference effects in GaN-HEMT power amplifier induced by microwave pulses were related to the deep-level traps inside the device. An isolator is a 2-port device that transmits signals only in one direction and prevents them from passing in the other^35^. Adding an isolator to the output of GaN-HEMT power amplifier can be used to improve this phenomenon.

## Conclusion

In summary, we have investigated the interference effects in GaN-HEMT power amplifier induced by microwave pulses. It was found that the normal output signal of the GaN-HEMT power amplifier may be disturbed when the microwave pulses were injected backward from the output end of device. The interference time can be on the order of milliseconds. The interference time and the maximum interference amplitude increase with the increase of the peak power of the reverse injection microwave pulses. Through analysis and comparative experiments, it is confirmed that trapping induced by deep traps is the main reason for this phenomenon. This finding is helpful for the protective strengthening of the GaN based devices.

## Data Availability

The datasets used and analysed during the current study are available from the corresponding author on reasonable request.
